# Cystoid macular oedema associated with Rosai–Dorfman disease: a case report

**DOI:** 10.1186/s12886-017-0538-8

**Published:** 2017-08-14

**Authors:** Rubens Camargo Siqueira, Antonio Augusto Velasco e Cruz

**Affiliations:** 1 Department of Ophthalmology, Otorhinolaryngology and Head and Neck Surgery, Ribeirao Preto School of Medicine, University of Sao Paulo, Sao Paulo, Brazil; 2 Rubens Siqueira Research Centre, Rua Saldanha Marinho 2815 Sala 42, Sao Jose do Rio Preto, Brazil

**Keywords:** Cystoid macular oedema, Rosai–Dorfman disease, Uveitis, Intravitreal triamcinolone

## Abstract

**Background:**

The aim of this study is to report a case of cystoid macular oedema (CME) associated with Rosai–Dorfman Disease (RDD).

**Case presentation:**

A 52-year-old male initially presented with a two-month history of a congested left eye. At presentation, visual acuity was 20/20 in the right eye and 20/80 in the left eye. Biomicroscopy showed conjunctival hyperaemia in the left eye with a slight elevation, suggesting a subconjunctival mass. Retinal fluorescein angiography and optical coherence tomography (OCT) revealed the presence of CME in the left eye. A clinical examination revealed nodular lesions in the ears and a lump in the subcutaneous tissue of the left arm. A biopsy of the subcutaneous lesion showed histological and immunohistochemical characteristics of RDD. The patient was treated with intravitreal triamcinolone (0.1 mL/4 mg). One month after treatment, there was complete regression of the oedema with a significant improvement in visual acuity to 20/20.

**Conclusions:**

This is the first reported case of RDD associated with cystoid macular oedema. Macular oedema responded to intravitreal treatment with triamcinolone.

## Background

Rosai–Dorfman disease (RDD), or sinus histiocytosis with massive lymphadenopathy, was first described by Destombes [1] in 1965. Four years later, Rosai and Dorfman [2] characterized RDD as a distinct clinicopathological disorder based on four cases. Patients with this rare and nonmalignant disorder often present with painless cervical lymphadenopathy, fever, and leukocytosis. RDD often manifests in the lymph nodes of the head and neck but may also be present in extranodal sites, such as the skin, conjunctiva, upper respiratory tract, and bone. Ocular involvement is relatively rare (8.5%) and usually manifests as lymphoproliferation in the soft tissues of the orbit and eyelid [3].

In the following report, we describe the first case of cystoid macular oedema (CME) associated with RDD.

## Case presentation

A 52-year-old male initially presented with a two-month history of congestion of the left eye.

**Fig. 1:**
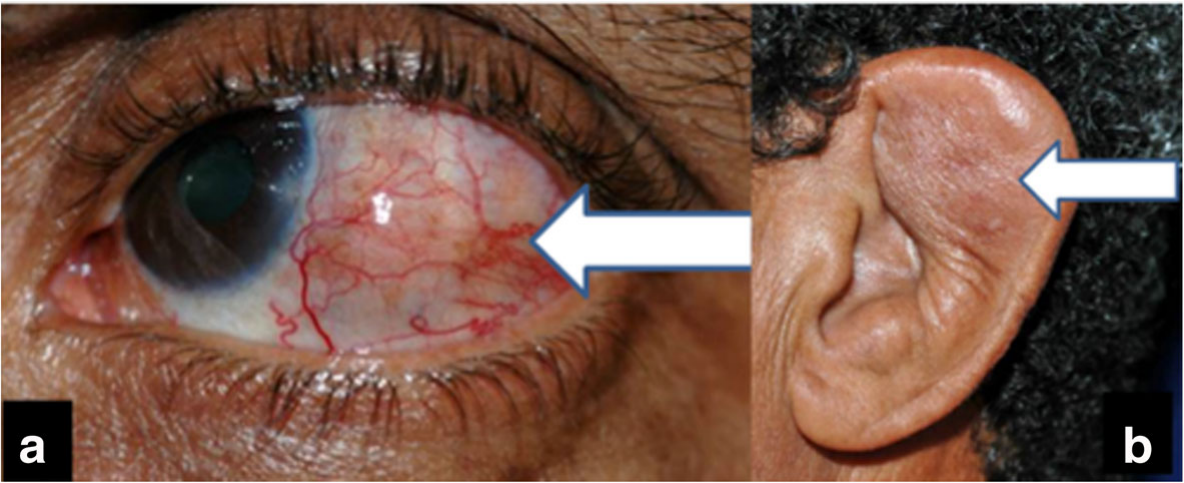
**a** Conjunctival hyperaemia with a slight elevation, suggesting a subconjunctival mass. **b** Nodular lesions in the ears

At presentation, he underwent a complete ophthalmic examination. Visual acuity was 20/20 in the right eye and 20/80 in the left eye. Biomicroscopy showed conjunctival hyperaemia in the left eye with a slight elevation, suggesting a subconjunctival mass (Fig. 1a), but no intraocular inflammatory reaction was noted. The bilateral intraocular pressures were both 10 mmHg. No signs of inflammatory cells were noted in the anterior chamber or the vitreous, and no signs of retinal vasculitis were identified on biomicroscopy and ophthalmoscopy examination with mydriasis. However, the presence of an abnormality in the foveal reflex in the left eye was observed. A clinical examination revealed nodular lesions in the ears (Fig. 1b) and a lump in the subcutaneous tissue of the left arm.

**Fig. 2:**
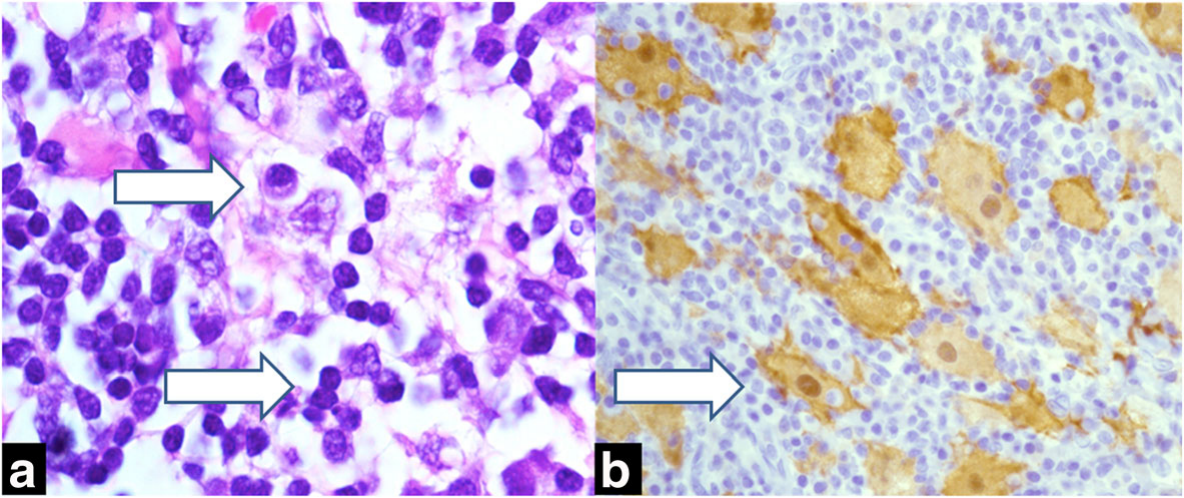
**a** Histopathological examination showing polymorphic plasma cell infiltration with lymphocytes, histiocytes, plasmocytes, and a few phagocytes (haematoxylin and eosin staining, 10×). **b** Large histiocytes exhibiting characteristic emperipolesis (haematoxylin and eosin staining, 40×)

**Fig. 3:**
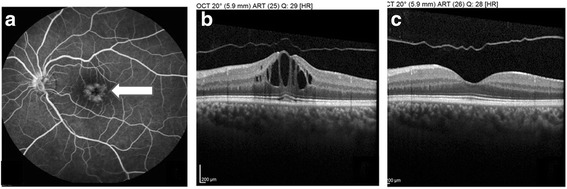
**a** Retinal fluorescein angiography showing cystoid macular oedema. **b** Optical coherence tomography (OCT) confirming cystoid macular oedema. **c** OCT showing complete regression of the oedema after intravitreal injection of triamcinolone

A biopsy of the subcutaneous lesion showed a cellular infiltrate in the superficial and deep dermis with histiocytes with phagocytized lymphocytes and plasma cells (Fig. 2a). A characteristic finding was the presence of lymphocytes engulfed within the histiocytic cytoplasm, a feature called emperipolesis (Fig. 2b) [1]. Immunohistochemically, the large histiocytes reacted strongly with antibodies to S-100 protein, CD68, and CD 20b. These three immunohistochemical markers are indicative of a mixed inflammatory or heterogeneous population of cells that are characteristic of RDD. Retinal fluorescein angiography in the left eye revealed petaloid leakage from the perifoveal retinal capillaries during the late phases without staining of the optic disc. Optical coherence tomography (OCT) revealed well-defined, intraretinal cystic areas of low reflectivity in the macula in the left eye, which are characteristic signs of cystoid macula oedema (CME). (Fig. 3b).

Due to the low visual acuity secondary to CME, the patient was treated intravitreally with triamcinolone (4 mg/0.1 mL). One month later, the patient showed significant improvement in visual acuity (20/20) and OCT showed complete regression of the oedema (Fig. 3c). The follow-up time was 6 months, and during this period, there was no recurrence of the oedema.

## Discussion and Conclusions

RDD (sinus histiocytosis with massive lymphadenopathy) often presents as a painless bilateral cervical lymphadenopathy with systemic symptoms. Consists of a rare disease, non-hereditary, benign histiocytic proliferative disorder. The Extranodal impairment have been reported in 28–43% of cases with rare ocular involvement [4].

The characteristic findings of RDD include emperipolesis, positive histiocyte staining for S100 and CD68, and negative staining for CD1a. There is no standardized protocol available for treatment of ocular RDD because of its rarity and the possibility of its spontaneous regression [2, 5]. Excisional biopsy is often performed for diagnosis and treatment, and there is usually no recurrence after this procedure [6].

Extradonal impairment has been reported in 28–43% of cases, with locations including the skin, upper respiratory tract, salivary glands, epidural space, bone, and ocular adnexa. Ocular manifestation is rare. In the largest series of 243 cases as described by Foucar in 1990, the eye was involved in 36 cases (8.5%) and was highly associated with nasal sinus involvement. Infiltration of the soft tissues of the orbit and eyelids was the most common manifestation of ocular involvement [5, 7]. In addition, infiltration of the lacrimal system, conjunctiva, subconjunctiva, cornea, uveal tract, and optic nerve were also reported [8–14].

Uveitis was present in three isolated cases of anterior uveitis (5,7,8,12) involving one patient with coexistent corneal infiltration [13], one patient with coexistent papilledema [14], one patient with bilateral subconjunctival masses [15], one patient with bilateral serous retinal detachment [16], and one patient with uveitic glaucoma [17].

To the best of our knowledge, this is the first report of macular oedema associated with RDD. The patient also presented with a subconjunctival mass and dermatologic abnormalities in the arm and ear. There was a prompt clinical response to intravitreal treatment with triamcinolone, suggesting a probable inflammatory cause of the macular oedema as previously reported in other forms of ocular involvement, such as chronic uveitis and uveitic glaucoma [12, 17].

The short follow-up time of 6 months was a limitation of this study, particularly with regard to drawing conclusions regarding the efficacy of long-term therapy.

In summary, RDD can be associated with CME; therefore, RDD patients should undergo routine eye examinations.

## Abbreviations

CME: Cystoid macular oedema
OCT: Optical coherence tomography
RDD: Rosai–Dorfman disease
